# A Novel Approach for a Toxicity Prediction Model of Environmental Pollutants by Using a Quantitative Structure-Activity Relationship Method Based on Toxicogenomics

**DOI:** 10.5402/2011/515724

**Published:** 2011-07-02

**Authors:** Junichi Hosoya, Kumiko Tamura, Naomi Muraki, Hiroki Okumura, Tsuyoshi Ito, Mitsugu Maeno

**Affiliations:** ^1^Energy and Environment Research Division, Japan Automobile Research Institute, 2530 Karima, Tsukuba, Ibaraki 305-0822, Japan; ^2^Graduate School of Science and Technology, Niigata University, 8050 Ikarashi-2, Nishi-ku, Niigata 950-2181, Japan

## Abstract

The development of automobile emission reduction technologies has decreased dramatically the particle concentration in emissions; however, there is a possibility that unexpected harmful chemicals are formed in emissions due to new technologies and fuels. Therefore, we attempted to develop new and efficient toxicity prediction models for the myriad environmental pollutants including those in automobile emissions. We chose 54 compounds related to engine exhaust and, by use of the DNA microarray, examined their effect on gene expression in human lung cells. We focused on IL-8 as a proinflammatory cytokine and developed a prediction model with quantitative structure-activity relationship (QSAR) for the IL-8 gene expression by using an *in silico* system. Our results demonstrate that this model showed high accuracy in predicting upregulation of the IL-8 gene. These results suggest that the prediction model with QSAR based on the gene expression from toxicogenomics may have great potential in predictive toxicology of environmental pollutants.

## 1. Introduction

It has been reported that the increase in ambient fine particulate matter (PM2.5, particulate matter with an aerodynamic diameter < or = 2.5 *μ*m) is associated with the mortality and morbidity from respiratory and cardiovascular diseases [1, 2]. Diesel exhaust particles (DEPs) are well known as one of the most important components of ambient PM2.5. The development of emission reduction technologies in recent years has produced considerable reduction in the particle concentration in diesel emissions; however, there is a possibility that unexpected toxic substances are produced in diesel emissions owing to new technologies and fuels [3]. Therefore, it is necessary to understand the toxicity of automobile emission consequential to these new technologies and fuels. Animal exposure studies can play an important role to evaluate the toxicity of environmental pollutants including those in automobile emissions. However, because the environmental pollutants are of great variety, it is impossible to understand in a cyclopaedic manner the toxicity of those only by such studies. Furthermore, an animal exposure study is a fairly long-term process and involves huge cost. In addition, the use of animal studies should be reduced from the view point of animal welfare. Therefore, there is a real need for the new approach for rational estimation of the toxicity of new environmental pollutants without the use of experimental animals.

In the field of toxicology, toxicogenomics has received a lot of attention in recent years. The DNA microarray method is a powerful tool for toxicogenomics to know the comprehensive gene expression change induced by various chemicals. By this technique, we can detect the toxic reaction to chemical compounds as changes in gene expression. It is said that a change in gene expression is “an early warning marker" of toxicity, because gene expression data provide useful information to predict the toxicity of chemicals before the phenotype is manifested [4–6]. On the other hand, the structure-activity relationship (SAR) approach, which elucidates the relationship between the chemical structure and biological activity of a compound of interest, has been in use over a long period of time. For the prediction of the toxicity including mutagenicity of candidate drugs for development, quantitative structure-activity correlation (QSAR) is utilized widely in the pharmaceutical industry [7–11]. Therefore, the fusion between toxicogenomics and QSAR may provide a high-accuracy toxicity prediction model for various chemical compounds.

Many studies have suggested that DEP induces the production of inflammatory markers in human lung epithelial cells [12, 13] and that exposure to diesel emissions augments endotoxin-induced pulmonary inflammation [14] and allergic airway inflammation in asthma model mice [15, 16]. Inflammatory cytokines and chemokines play important roles in these inflammatory responses [17]. In particular, IL-8 is a well-known inflammatory cytokine involved in allergic inflammation [18], and its expression is upregulated by exposure of animals to diesel emissions or to treatment with DEP *in vitro* [19–23]. Although many reports suggest that diesel emission affects allergic responses, it is not clear what components of DEP are responsible for it. 

In this study, we focused on the relationship between IL-8 gene expression and DEP and sought to develop, by using the methodologies of toxicogenomics and QSAR, a prediction model for IL-8 gene expression elicited by various chemicals found in diesel exhaust. To this end, we (1) analyzed the gene expression in A549 cells (human epithelial cell line) treated with 54 chemicals related to diesel emissions by using the DNA microarray method, (2) constructed a prediction model of IL-8 gene expression by using information about the physicochemical characters of these 54 chemicals and IL-8 gene expression and (3) validated the prediction model of IL-8 gene expression according to known data from previous reports.

## 2. Materials and Methods

### 2.1. DEP and Chemicals

The diesel exhaust particles, SRM 2975 (Industrial Forklift), were purchased from the National Institute of Standards and Technology (Gaithersburg, Md, USA). Other chemicals were obtained from Wako Pure Chemical Industries (Osaka, Japan).

### 2.2. Cell Culture

The A549 cell line was purchased from the American Type Culture Collection (CCL 185 line; Rockville, Md, USA). Cells were kept at 37°C in a humidified incubator under 5% CO_2_ in air and grown in DMEM culture medium containing 10 *μ*g/mL gentamicin supplemented with 10% FBS until they had reached 80–90% confluence.

### 2.3. Treatment with DEPs and Chemicals

DEP and various chemicals were dissolved and sonicated in DMSO. A549 cells (1 × 10^6^) were seeded into each of several dishes. Two days after the seeding, the cells were exposed to DEP or various chemicals for 4 hours. Final concentrations were 1 *μ*M and 10 *μ*M for chemicals and 30 *μ*g/mL and 100 *μ*g/mL for DEP. These concentrations were decided based on the results of the cytotoxicity examination (data not shown). Control cells were treated with the same concentration of DMSO. After the exposure, total RNA was extracted from cells by using an RNeasy mini kit (QIAGEN, Hikden, Germany) according to the manufacturer's protocol, eluted with RNase-free water, and stored at −80°C prior to use. RNA concentrations were determined with a spectrophotometer (GeneQuant, Amersham Biosciences, Piscataway, NJ, USA) and analyzed for quantity and quality by using a bioanalyzer (Agilent Technologies, USA).

### 2.4. DNA Microarray Experiment and Data Analysis

Total RNA was used for fluorescently labeled cRNA synthesis with an Agilent Quick Amp Labeling Kit (Agilent Technologies, USA), and Cy3-labeled cRNA was combined with and hybridized to Agilent 4 × 44 K Human Oligo Microarrays (Agilent Technologies, USA) according to the manufacturer's protocol. After hybridization, the slides were washed and scanned with an Agilent microarray scanner. The scanned images for each slide were analyzed by using Feature Extraction software version 9.5.3.1 (Agilent Technologies, USA). The obtained data were then analyzed by using GeneSpring GX 10.0 software (Agilent Technologies, USA). The data were normalized by the per chip normalization method, and filtering of the data was performed by using flags (present, absent, and marginal).

### 2.5. Construction and Validation of the Prediction Model

The 54 chemicals were classified into 2 groups based on the gene expression of IL-8. One was the upregulation class and the other, the downregulation class. Successively, 372 physicochemical descriptors of the chemicals were calculated by the use of ADMEWORKS (Fujitsu, Japan). Then, some of these descriptors related to IL-8 expression were chosen, and a prediction model was constructed by using the ADMEWORKS.

## 3. Results and Discussion

### 3.1. Gene Expression Analysis of the 54 Chemicals

**Figure 1:**
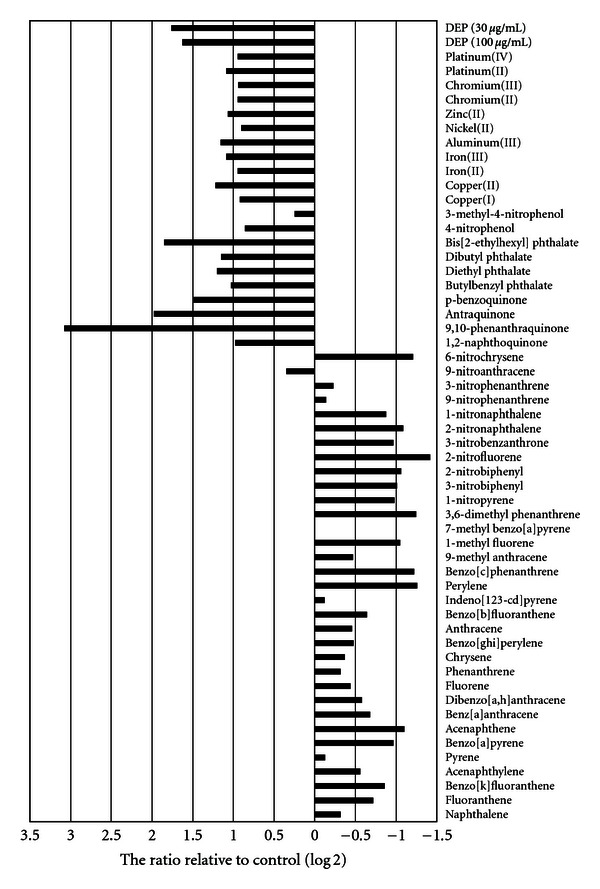
IL-8 gene expression induced by the 54 chemicals and DEP in A549 cells. The A549 cells were exposed separately to each of the 54 chemicals or to DEP for 4 hours. Changes in the expression level of the IL-8 gene were measured by using the DNA microarray described under “Materials and methods." Data are presented as relative change fold (log2) to control cells. The results are based on 1 experiment.

We analyzed tens of thousands of genes by DNA microarray and focused on IL-8 as a proinflammatory cytokine. IL-8 gene expression in A549 cells treated separately with each of the 54 chemicals is shown in Figure 1. DEP upregulated its expression, a result supported by previous reports [24, 25]. Because it appeared that IL-8 gene expression depended on the type of materials, we thought that the chemical structure of the materials was important for the gene expression. IL-8 was downregulated by most PAHs and nitroarenes. On the other hand, it was upregulated by quinones, phthalates, nitrophenols, and metals. However, it was earlier reported that IL-8 is upregulated by PAHs in human lung epithelial cells [26, 27]. This discrepancy may have been caused by the difference in the experimental conditions such as treatment time between those reports and our study. Because we confirmed that IL-8 gene expression was most strongly upregulated by DEP treatment for 4 hours in our experimental environment in preliminary experiments (data not shown), we fixed the treatment time at 4 hours.

### 3.2. Construction of the Prediction Model of IL-8

We classified the 54 chemicals into 2 classes, that is, upregulation class and downregulation class, by using ADMEWORKS, which is a chemical compound toxicity prediction system, and the IL-8 gene expression data obtained from the DNA microarray. As a result, the following model was built:

(1) $$\begin{array}{cc} y & =-0.57\left[\text{WTPT}3\right]+0.44\left[\text{MOLC}4\right]+0.31\left[\text{V}5\text{CH}\right]\\ & \;\;+0.30\left[\text{SYMM}2\right]+0.19\left[\text{S}3\text{C}\right]-0.15\left[\text{CRB}\_\text{LEADL}\right]\\ & \;\;-0.02\;\left[\text{OPERA}\_\text{RULEI}\right], \end{array}$$

*y* > 0, downregulation, *y* < 0, upregulation.

Table 1 shows the 7 descriptors used in this prediction model and their degree of contribution to the IL-8 gene expression and Table 2, the values of these descriptors of all 54 chemicals. If the absolute value of the contribution degree is large, the chemical is closely linked to variability of IL-8 gene expression in A549 cells. Furthermore, a positive value for the contribution degree is related to downregulation of the cytokine and a negative one, to upregulation of it. We thought that the IL-8 gene expression in A549 cells treated with any chemicals could be predicted by this model from knowing the chemical structures. The rate of classification of the 54 chemicals except for DEP by this model was 92%.

In this prediction model, WTPT3, CRB_LEADL, and OPERA_RULEI were related to upregulation of IL-8 gene expression. Since the contribution degree of WTPT3 was the highest, we considered WTPT3 to be the most important descriptor related to upregulation of IL-8 gene expression. WTPT3 refers to the sum of atom indexes for all heteroatoms. The atom index means the number of the bond order between arbitrary atom pairs; in other words, it indicates the structural environment around the heteroatoms. In our analysis, the IL-8 gene expression in the A549 cells was downregulated by PAHs and upregulated by quinones, phthalates, and metals. Reflecting this, the WTPT3 values of the quinones, phthalates, and metals were larger than those of the PAHs. As PAHs are chemical compounds that consist of fused aromatic rings and do not contain heteroatoms, we considered these results to be reasonable. CRB_LEADL means the count of rotatable bonds. CRB_LEADL values for the phthalates were high. The numerousness of rotatable bonds indicates that such a molecule can assume the shape of various stereoisomers. In fact, the phthalates are known to form several stereoisomers. Since the IL-8 gene expression was strongly upregulated by phthalates in our analysis, the number of stereoisomer may be important for upregulation of the IL-8 gene expression. OPERA_RULEI is a value that reflects the “rule of five” of Lipinski, which is related to oral bioavailability [28]. The significance of it in this model, based on the data from the *in vitro* assay, is unknown. Since there was no great distinction among chemicals in terms of their OPERA_RULEI value, the contribution degree of this descriptor might be low. We consider that the role of this descriptor in the upregulation of IL-8 was complementary. 

MOLC4, V5CH, SYMM2, and S3C were related to downregulation of IL-8 gene expression. Among these descriptors, MOLC4 showed the highest contribution degree; therefore, it could be the most important descriptor related to the downregulation of IL-8 gene expression. MOLC4 refers to the total of the pass weight about atom pairs that are 2 bonds in distance from one another. The term “pass” means the shortest distance between 2 arbitrary atoms, and the pass weight means the weighted value of the pass. The MOLC4 values of PAHs and nitroarenes, whose compounds downregulated IL-8 gene expression, tended to be high. In particular, the MOLC4 values of the chemicals that had more than 5 benzene rings, such as Benzo[a]pyrene, Dibenzo[a,h]anthracene, Benzo[ghi]perylene, Indeno[123-cd]pyrene, and 7-methyl benzo[a]pyrene, were high; the average of their MOLC4 values was 5.436. V5CH means the total of the pass weight about atom pairs that are 5 bonds away from each other and S3C, the total of the pass weight about a 3rd order cluster. SYMM2 refers to the geometrical symmetry of the pass. A low value for SYMM2 means that the molecular symmetric property is large. For some chemicals that downregulated the gene expression, their V5CH values were equal to or less than 0.1. Furthermore, there was no remarkable difference among the chemicals regarding SYMM2 and S3C, either. As the contribution degree of these descriptors was low, we considered that these descriptors in downregulation of IL-8 were complementary. 

In terms of IL-8 gene expression, WTPT3 and MOLC4 are the most important descriptors, showing the topological information about the chemicals. In brief, the property of unchangeability of the molecule may be important for affecting IL-8 gene expression.

### 3.3. Validation of the Prediction Model

The prediction model of IL-8 gene expression was validated by previous reports indicating that some chemicals changed the IL-8 gene expression level in A549 cells in the same manner as found in this present study. It is generally thought that IL-8 is related to inflammation [29] or oxidative stress [30]. Therefore, it is thought that the IL-8 may be upregulated by proinflammatory compounds and oxidants. Therefore, we chose chlorobenzene [31], sodium sulfite [32], and sphingosine-1-phosphate [33] as proinflammatory compounds, and paraquat [34] as an oxidant (Table 3).

On the other hand, it is generally thought that IL-8 expression may be downregulated by anti-inflammatory compounds and antioxidants. Therefore, we chose dexamethasone [35] as an anti-inflammatory compound and *β*-carotene [36] and theaflavin [37] as antioxidants (Table 4). In addition, it is well known that NF-*κ*B, a transcription factor, plays an important role in inflammation [38]. Since it is reported that isohelenin, an NF-*κ*B inhibitor [39, 40], down-regulates IL-8 at the mRNA level in A549 cells [41], we chose it as an NF-*κ*B inhibitor for validation of the prediction model of IL-8 (Table 4). The results of the evaluation are shown in Tables 3 and 4. 

The prediction model of IL-8 for all compounds that we chose from previous reports showed 75% accuracy. The prediction of upregulation was 100% accuracy and that of downregulation was 50% accuracy in this model. There was no discrepancy between previous data and prediction of upregulation of IL-8. However, although it was previously reported that IL-8 gene is downregulated by dexamethasone and theaflavin, our model predicted upregulation by those compounds. High values of WTPT3 for those compounds may have confused the prediction of IL-8 gene expression, and we suspect that WTPT3 might have been overestimated in our model. On the other hand, it is reasonable that the values of MOLC4, which is thought to contribute to downregulation, were high for dexamethasone, theaflavin, *β*-carotene, and isohelenin. These results suggest that there is still room for improvement of the model formula to be able to reflect downregulation of IL-8 even when WTPT3 is high. In future, it will be necessary to accumulate data by analyzing many compounds with diverse structures and to continuously rebuild a prediction model to obtain higher accuracy.

### 3.4. The Necessity of Prediction Model

Until now, there have not been many studies evaluating the toxicity of chemicals by means of *in silico* and *in vitro* assays. A US Environmental Protection Agency (EPA) report notes the need to leverage *in vitro* assays using human cell lines and computational toxicology in their “Strategic Plan for Evaluating the Toxicity of Chemicals" [42]. Although our toxicity prediction model, which fuses toxicogenomics and QSAR, is still in the trial phase, it may be a step in the right direction for future assessment of the toxicology of environmental pollutants.

## 4. Conclusions

The results of this study showed that the construction of a new toxicity prediction model for environmental pollutants based on QSAR and gene expression data might be useful to understand the various biological reactions about not only mutagenicity as in traditional toxicology but also inflammation and other toxicological responses.

## Figures and Tables

**Table 1:** List of the descriptors related to IL-8 gene expression.

Descriptor	Abbreviation	Contribution degree	Relationship with IL-8 gene expression
Sum of atom indexes for all heteroatoms	WTPT3	−0.57	upregulation
Path-2 molecular connectivity	MOLC4	0.44	downregulation
5th order chain MC Valence	V5CH	0.31	downregulation
Geometrical symmetry	SYMM2	0.30	downregulation
3rd order cluster MC Simple	S3C	0.19	downregulation
Count of rotatable bonds	CRB_LEADL	−0.15	upregulation
The rule based on Lipinski's rule	OPERA_RULEI	−0.02	upregulation

**Table 2:** Descriptor values of the 54 chemicals.

Material	WTPT3	MOLC4	V5CH	SYMM2	S3C	CRB_LEADL	OPREA_RULEI
*Polyaromatic hydrocarbon* *(PAH) *							
Naphthalene	0.00	2.23	0.00	0.30	0.33	0.00	1.00
Fluoranthene	0.00	4.13	0.03	0.38	0.82	0.00	1.00
Benzo[k]fluoranthene	0.00	5.21	0.03	0.30	1.16	0.00	0.00
Acenaphthylene	0.00	3.00	0.04	0.50	0.61	0.00	1.00
Pyrene	0.00	3.93	0.00	0.25	0.89	0.00	1.00
Benzo[a]pyrene	0.00	4.97	0.00	0.25	1.16	0.00	0.00
Acenaphthene	0.00	3.30	0.06	0.50	0.61	0.00	1.00
Benz[a]anthracene	0.00	4.35	0.00	0.28	0.94	0.00	1.00
Dibenzo[a,h]anthracene	0.00	5.38	0.00	0.18	1.21	0.00	0.00
Fluorene	0.00	3.49	0.04	0.54	0.61	0.00	1.00
Phenanthrene	0.00	3.26	0.00	0.36	0.61	0.00	1.00
Chrysene	0.00	4.35	0.00	0.28	0.94	0.00	1.00
Benzo[ghi]perylene	0.00	5.59	0.00	0.18	1.38	0.00	0.00
Anthracene	0.00	3.31	0.00	0.29	0.67	0.00	1.00
Benzo[b]fluoranthene	0.00	5.17	0.03	0.40	1.09	0.00	0.00
Indeno[123-cd]pyrene	0.00	5.83	0.03	0.45	1.38	0.00	0.00
Perylene	0.00	4.93	0.00	0.20	1.10	0.00	0.00
Benzo[c]phenanthrene	0.00	4.31	0.00	0.33	0.88	0.00	1.00
9-methyl anthracene	0.00	3.69	0.00	0.27	0.80	0.00	1.00
1-methyl fluorene	0.00	3.93	0.04	0.57	0.81	0.00	1.00
7-methyl benzo[a]pyrene	0.00	5.41	0.00	0.24	1.37	0.00	0.00
3,6-dimethyl phenanthrene	0.00	4.27	0.00	0.38	1.18	0.00	1.00

*Nitroarene*							
1-nitropyrene	7.53	4.26	0.00	0.42	1.33	1.00	1.00
3-nitrobiphenyl	7.53	3.09	0.00	0.67	0.83	2.00	1.00
2-nitrobiphenyl	7.55	3.06	0.00	0.73	0.77	2.00	1.00
2-nitrofluorene	7.53	3.85	0.04	0.63	1.11	1.00	1.00
3-nitrobenzanthrone	10.04	4.79	0.00	0.38	1.45	1.00	1.00
2-nitronaphthalene	7.52	2.59	0.00	0.62	0.83	1.00	1.00
1-nitronaphthalene	7.54	2.56	0.00	0.85	0.77	1.00	1.00
9-nitrophenanthrene	7.52	3.60	0.00	0.41	1.04	1.00	1.00
3-nitrophenanthrene	7.52	3.63	0.00	0.41	1.11	1.00	1.00
9-nitroanthracene	7.54	3.61	0.00	0.41	1.05	1.00	1.00
6-nitrochrysene	7.52	4.63	0.00	0.43	1.32	1.00	1.00

*Quinone*							
1,2-naphthoquinone	4.97	2.61	0.00	0.58	0.73	0.00	1.00
9,10-phenanthraquinone	4.98	3.75	0.00	0.31	0.93	0.00	1.00
Antraquinone	5.03	3.75	0.00	0.31	0.93	0.00	1.00
p-benzoquinone	4.93	1.47	0.00	0.38	0.58	0.00	1.00

*Phthalate*							
Butylbenzyl phthalate	10.56	5.10	0.00	0.61	0.95	9.00	1.00
Diethyl phthalate	10.46	2.99	0.00	0.69	0.74	6.00	1.00
Dibutyl phthalate	10.63	4.52	0.00	0.50	0.74	10.00	1.00
Bis[2-ethylhexyl] phthalate	10.63	7.58	0.00	0.43	1.15	16.00	0.00

*Nitrophenol*							
4-nitrophenol	9.78	1.69	0.00	0.60	0.79	1.00	1.00
3-methyl-4-nitrophenol	9.83	2.15	0.00	0.73	0.99	1.00	1.00

*Metal*							
Copper (I)	4.00	0.00	0.00	0.50	0.00	0.00	1.00
Copper (II)	6.83	0.52	0.00	0.67	0.00	0.00	1.00
Iron (II)	6.83	0.52	0.00	0.67	0.00	0.00	1.00
Iron (III)	9.46	3.96	0.00	0.50	0.58	0.00	1.00
Aluminum (III)	9.46	3.96	0.00	0.50	0.58	0.00	1.00
Nickel (II)	6.83	0.52	0.00	0.67	0.00	0.00	1.00
Zinc (II)	6.83	0.52	0.00	0.67	0.00	0.00	1.00
Chromium (II)	6.83	0.52	0.00	0.67	0.00	0.00	1.00
Chromium (III)	9.46	3.96	0.00	0.50	0.58	0.00	1.00
Platinum (II)	6.83	0.52	0.00	0.67	0.00	0.00	1.00
Platinum (IV)	12.00	4.07	0.00	0.40	2.00	0.00	1.00

**Table 3:** List of chemicals up-regulating IL-8 gene expression and prediction results.

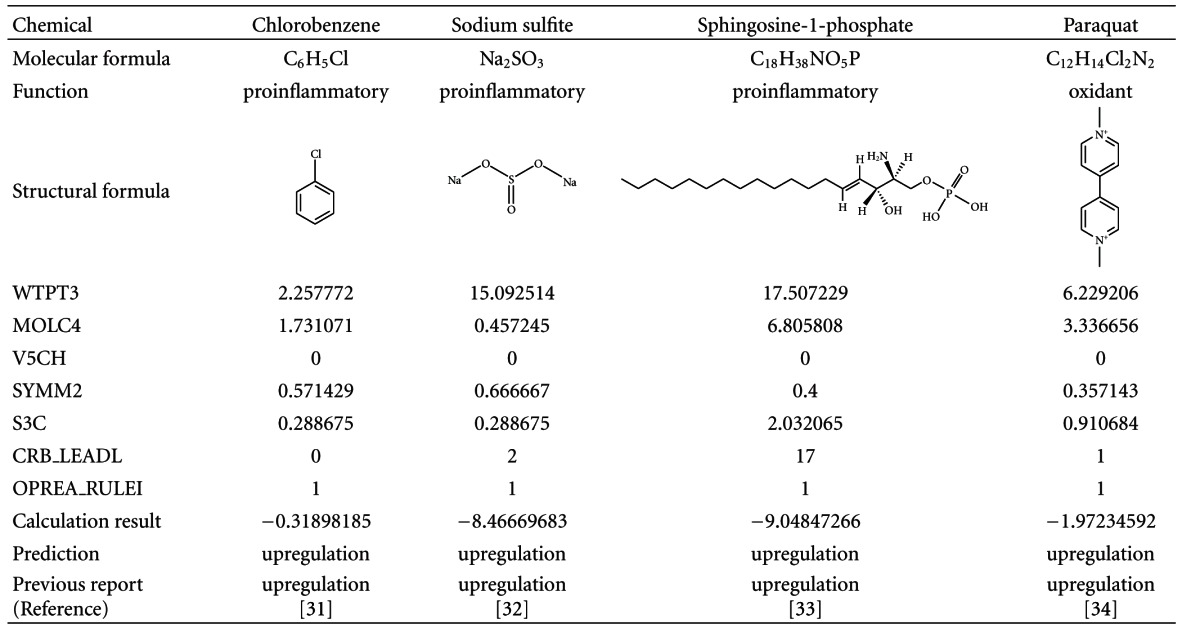

**Table 4:** List of chemicals down-regulating IL-8 gene expression and prediction results.

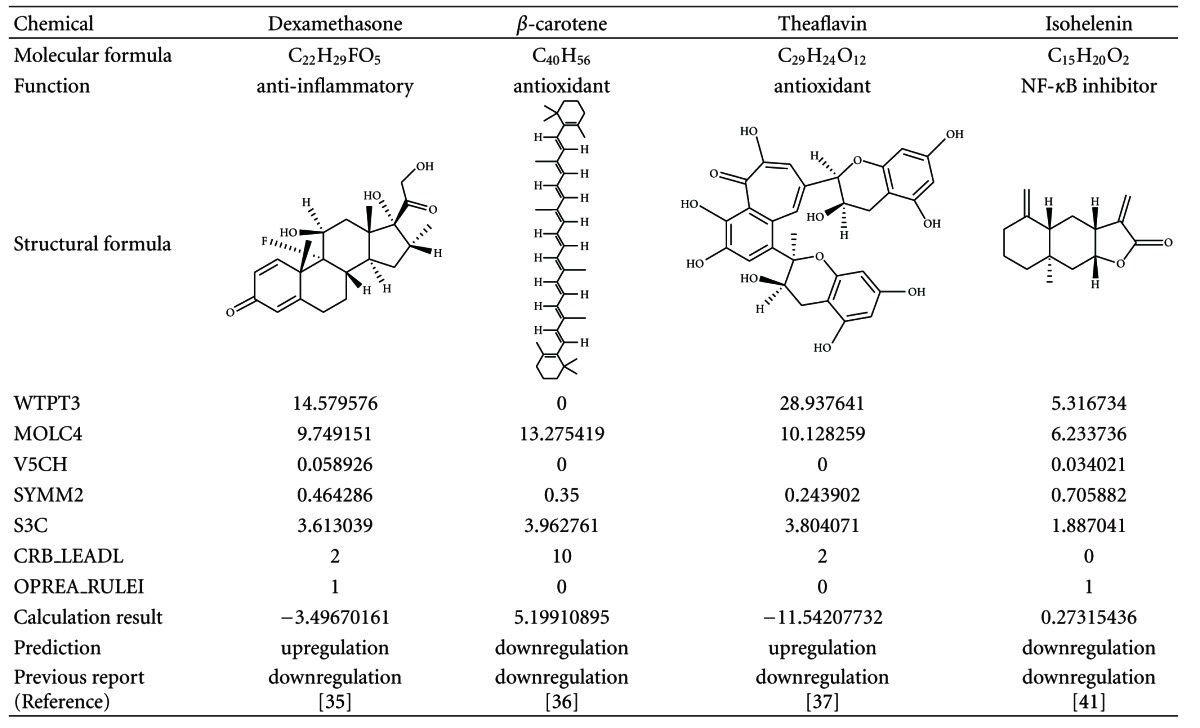

## Abbreviations

QSAR: Quantitative structure-activity relationship
DEPs: Diesel exhaust particles.
